# Brief Research Report: “Nothing About Us, Without Us”: A Case Study of the Outer Sanctum Podcast and Trends in Australian Independent Media to Drive Intersectional Representation

**DOI:** 10.3389/fspor.2022.871237

**Published:** 2022-05-09

**Authors:** Kasey Symons, Sam Duncan, Emma Sherry

**Affiliations:** School of Business, Law and Entrepreneurship, Swinburne University of Technology, Hawthorn, VIC, Australia

**Keywords:** sports media, new media, *The Outer Sanctum* Podcast, intersectionality, diversity, podcasting

## Abstract

Alternative and independent sports media platforms create custom content that reflects a diversity of voices and representation of athletes, sports and issues that are not covered in meaningful ways in traditional sports media. While these new media outlets often set out to redress the lack of diversity and intersectional approaches to traditional sports media, they are also seeking ways to drive even more change. This in an interesting and important movement to interrogate as these platforms are not only predominantly unfunded, passion projects created by those marginalized groups. This brief research report provides a case study into this emerging alternative media space and its impact in driving change in an ever-evolving sports media landscape. We also discuss the problematic nature of intersectional-redressing work falling on those who still occupy the margins. This report uses a case study of an independent Australian rules football platform, *The Outer Sanctum* podcast, to focus on these key areas. The case study investigates how this outlet has worked to increase the visibility and profile of marginalized and underrepresented voices discussing football in new ways. It follows their journey as they have taken steps to improve their own diversity, enacting their mantra “nothing about us, without us,” and proactively becoming more intersectional in their content producing journey. This research report will present key findings from the work of this media outlet to drive change and point to the learnings mainstream media can adopt to meaningfully embed intersectional approaches to sports media as core business.

## Introduction

The emergence of digital media and its capacity to offer alternative and accessible ways to create and distribute content has seen great shifts in the modern media landscape with sports media no exception (Duncan, 2020). In the sports media space, specific content is now being created by passionate fans, bloggers and aspiring journalists, adding to an already oversaturated field of sports news, opinion and analysis. Importantly, new digital media offerings are proving productive for those from traditionally marginalized communities to add their voices to the sports media landscape. These content creators draw on easy to use and often free platforms such as podcasts, blogs, websites, newsletters and social media accounts to add their voices to sports media. They challenge what is perceived to be a continuation of a media model that excludes coverage of women's sports (Symons et al., 2021), disability sports and niche sports, a model that silences, or ignores, complicated issues within sport and only offers platforms to voices that are mostly white, cisgender and heterosexual (cishet) men (Cooky et al., 2015; Sherwood et al., 2017). These creators seek to engage with diverse sports, diverse communities, and engage with challenging issues with nuance and appropriate cultural understanding.

Alternative and independent sports media platforms create custom content that reflects a diversity of voices and representation of athletes, sports and issues that are not covered in meaningful ways in traditional sports media. While these new media outlets often set out to redress the lack of diversity and intersectional approaches to traditional sports media, they are also seeking ways to drive even more change as they work on becoming more intersectional and representative, not only in the content they produce but also in the voices they include and amplify. This in an interesting and important movement to interrogate as these platforms are not only predominantly unfunded passion projects created by those marginalized groups who have not been afforded opportunities in traditional media spaces, but continue to produce unpaid work in the hope of driving change from a bottom-up approach.

The momentum in alternative sports media digital content creation suggests there is a significant appetite for intersectional diversity in sports media, so much so that the platforms and content creators that already sit outside of mainstream sports media because of its traditional lack of diversity, are themselves proactively becoming more reflective of their smaller, niche audiences in their own practices.

This brief research report provides a preliminary investigation into the strategies some alternative and independent sports media platforms are employing to be more intersectional and inclusive in their content production, the challenges that exist for independent sports media to do so, and the impacts these platforms can have within communities while challenging the practices and systemic newswork routines (Sherwood et al., 2017) of mainstream sports media.

## Literature Review

Intersectionality in the context of the Australian sports media landscape is an emerging and underdeveloped area of research. While intersectionality aims to make visible the interconnected nature of social categories such as gender, race, class, age and sexuality and how these interact to reproduce social inequalities (Collins and Bilge, 2020), gender identity has generally been prioritized by sports media academics, often without considering that gender inequality can be mediated by other systems of discrimination (Bruce, 2016).

In her paper about sportswomen and media representation, Bruce (2016) highlights that “because gender is privileged as *the* defining difference, other axes of identity are not always acknowledged as relevant,” yet in reality, sports bodies and sports media organizations are “never only gendered, they are infused by meanings relating to multiple intersecting relations of power” (Hills and Kennedy, 2009). As a result, the “sportswoman” discussed in many research studies is most likely to be “white, elite, heterosexual, able bodied, middleclass” (Bruce, 2016).

Studies conducted over the last 30 years have consistently found the representation of women working in sports newsrooms is close to 10% (Henningham, 1995; Whiteside and Hardin, 2006; Nicholson et al., 2011), while women's sports coverage accounts for <10% (Lumby et al., 2014; Cooky et al., 2015, 2021). Further, when women's sport is covered, rather than being prioritized, it is generally treated as secondary, or peripheral to men's sport. This subordinate treatment of women's sports by newsrooms is highlighted by Cooky et al. (2021) who found that only two percent of 251 news broadcasts they analyzed led their bulletin with a story about women's sport.

Additionally, sports media professionals are more likely to be white men and “the sports media newsroom has been a particularly male-dominated, hegemonic environment” (Cooky et al., 2015, 2021; Sherwood et al., 2017). Sherwood et al. (2017) highlighted sports newsrooms are generally run by men who prioritize men's sport, believing it to be more newsworthy and that the majority of sports news consumers want to hear about men's sport. Of course, male dominated newsrooms are not confined to the Australian sports media landscape. The most recent *Sports Media Gender and Racial Report Card* (Lapchick, 2018) revealed 91.5% of sports editors and 90.2% of assistant sports editors in the United States were male. In their study of women's sports representation in US televised sports news and highlights shows, Cooky et al. (2015) found that over 95 percent of sports news and highlight anchors, and 96 percent of “sports analysts,” were male. When the study was expanded to include ancillary reporters on sports shows, female representation grew slightly to 14.4%. A 2019 follow up study by Cooky et al. (2021, p. 367) found little had changed from their previous research, highlighting “the majority of anchors, co-anchors, and ancillary announcers/ analysts featured were white men.”

The representation of other minority groups is similarly unbalanced with 88 per cent of Australian sport journalist being Australian born (Nicholson et al., 2011). Three-quarters of Australian mainstream media have an Anglo-Celtic background, while only 6 percent have an Indigenous or non-European background; the majority of those from a culturally diverse background believe this is a barrier to career progression (Arvanitakis et al., 2020).

Yet, there are signs of change within the Australian sports media industry regarding the representation of alternative voices (Nicholson et al., 2011; Sherwood et al., 2017; Sherwood, 2019). The advent of social and digital media platforms has disrupted hegemonic narratives (Bruce, 2016; Thorpe et al., 2017) and democratized the sports media landscape (Miah, 2017). Digital media has empowered audiences to become content creators and share their experiences. This form of citizen journalism has provided a voice for underrepresented groups to create independent digital sports media products, including blogs, websites and podcasts.

Research has highlighted how digital platforms can be used to advocate for gender equality, provide a platform to discuss and celebrate female, trans and non-binary athletes and sport, discuss important intersectional issues and stories that are largely ignored by mainstream media outlets, and legitimize women talking about both men's and women's sport (Antunovic and Linden, 2014; Sherwood, 2019).

There has also been a noticeable, albeit modest, shift in the presentation of sport in mainstream media. Perhaps spurred by the introduction and popularity of new women's professional and semi-professional sporting leagues in Australia, television networks have introduced more women and non-binary voices into their coverage of popular sports, including male dominated sports such as men's cricket and Australian rules football. However, while there are now more women and non-binary sports media professionals in more prominent media roles, they remain a distinctly underrepresented voice in mainstream coverage of sport (Sherwood, 2019).

Rather, it is the digital sphere that is best providing alternative independent media producers with the opportunity to push the boundaries and truly represent diverse communities (Sherwood, 2019). Many of these products have proven to be extremely successful, generating large audiences and stimulating interaction from diverse, vibrant, engaged communities. Some, such as *The Outer Sanctum* podcast have previously been “picked up” by mainstream media organizations and incorporated into their suite of products and channels, however there are significant challenges associated with producing alternative independent media (Sherwood, 2019).

In today's ultra-competitive media landscape, it is extremely difficult to monetise alternative online media products, particularly those that are not supported by larger mainstream media companies. Those producing alternative media generally “use significant unpaid labor to produce their sports media products” which can “have a high potential for burnout” (Gleeson, 2016; Sherwood, 2019). Those who are supported by mainstream media face their own challenges. Given the mainstream media economy and sports-media-business nexus is still largely built on the widespread appeal of sport, most mainstream media organizations continue to place a significant emphasis on producing content that attracts and engages mass, mainstream audiences (Evans et al., 2013; Nicholson et al., 2015; Duncan, 2020). Thus, due to the commercial importance of ratings and engagement metrics to attract advertisers and other funding (Nelson, 2019), it is possible mainstream media organizations will attempt to influence the editorial content of alternative media products to ensure they appeal to a broader audience. Alternatively, the hosts and producers of alternative media products may be encouraged to be more controversial or sensationalist in order to create headlines and invoke a reaction from mainstream consumers, thus ensuring the product is topical and newsworthy (Nelson, 2019). These practices, while potentially commercially advantageous, can corrupt the vision, spirit and purpose of the product.

Furthermore, while many alternative independent media products have been created to provide a voice for underrepresented communities, far fewer products have sought to consciously represent the intersectionality of gender, sexuality, race, age, social class, sport ableness and other forms of disadvantage and discrimination (Bruce, 2016). Thus, while the digital age has given rise to alternative voices representing minority groups in sport, there remains a gap in sports media that considers and addresses broader intersections of discrimination, disadvantage and power relations.

One media product that has actively developed and evolved to become more intersectional is *The Outer Sanctum* podcast.

This brief research report uses *The Outer Sanctum* podcast as a case study to understand the role of alternative, independent media through an intersectional lens. To research the creation, impact and challenges of *The Outer Sanctum* podcast this report draws on the existing literature of Australian based alternative and independent sports media platforms, the rise of digital media and state of play for intersectional sports media coverage alongside publicly available records (media articles, interviews, essays and audio recordings) that reference and/or discuss *The Outer Sanctum* podcast or were produced by members of *The Outer Sanctum* podcast. The case study brings together these elements to create a narrative of *The Outer Sanctum* podcast's inception, growth in popularity, opportunities, proactive moves toward diversity and inclusion and the ongoing challenges in this space. By viewing the inception and growth of The Outer Sanctum through an intersectional framework, we are able to interpret and understand the opportunities, impact, challenges and limitations that exist for media that consciously represent minority communities and intersections of discrimination. Through applying intersectionality theory to a case such as *The Outer Sanctum* podcast, a preliminary investigation, which raises important discussions, issues and questions, is formed, which can be drawn upon to guide future research.

## Case Study: *The Outer Sanctum* Podcast

*The Outer Sanctum* podcast is one example of an alternative independent media product that has grappled with representing the voices of diverse communities in sport and has actively worked to become more intersectional.

*The Outer Sanctum* Podcast was launched in 2016 by six Melbourne-based women. Sisters Emma, Lucy and Felicity Race, Nicole Hayes, Kate Seear and Alicia Sometimes first bonded together as a group through their mutual support of their Australian rules football team, the Hawthorn Football Club. The group of six wanted to extend their conversations about football to a wider audience, believing their approach to discussing the game they loved as women was something lacking from a largely male-dominated mainstream sports media (O'Halloran, 2018).

As Sherwood (2019) details in their work on women independent sports media producers in Australia, *The Outer Sanctum* podcast team were, and are, part of an emerging trend in alternative sports media that is utilizing the growth of accessible digital media.

In Australia, a growing cohort of women is taking advantage of the digital age by using websites, podcasts and digital and social media to produce independent sports media products. As stated above, these have attracted unique audiences and have seemingly been legitimized as media products. (Sherwood, 2019, p. 184)

It did not take long for the podcast to gain attention as it grew a highly engaged and loyal following of fans who were also seeking a different football discourse to that of mainstream media; their conversations also called attention to the darker misogyny of sports media, putting the podcast in the spotlight (Australian Broadcasting Corporation (ABC)., 2016).

Sherwood details that the podcast “had been live for only a few weeks in 2016 when it made headlines” (Sherwood, 2019, p. 184).

They called out comments made on radio by [former] Collingwood Australian Football League (AFL) club president and AFL commentator Eddie McGuire, where he discussed drowning prominent female football journalist Caroline Wilson. However, before the intervention of *The Outer Sanctum* Podcast, the mainstream media had largely ignored his comments. The six women's subsequent condemnation of McGuire's and others' words and the misogynistic environment of professional sports became the subject of widespread criticism, and McGuire and others were ultimately forced to apologize for their comments. (Sherwood, 2019, p. 184)

Co-founder Nicole Hayes wrote of the experience for the *Griffith Review* to navigate the surprise and shock that the group encountered during this time.

While none of us had the slightest notion of the scale of controversy our podcast would engender, when we first formed *The Outer Sanctum* we knew it would give voice to stories not ordinarily heard. The point was to have fun while creating a safe and welcoming space for everyone; but mostly, we wanted to discuss the issues that matter to us, as women, as parents, as people—though always, ultimately, as fans of the game. (Hayes, 2016)

While the women experienced negative and deeply sexist media attention, most notably from former co-host of *The Footy Show* (former popular long-running Australian sports panel show) Sam Newman who referred to the women as “cowardly excrement” (see Hayes, 2016), the subsequent catapult into Melbourne's highly saturated football media for the show was rapid and somewhat welcomed by traditional mainstream media outlets who were struggling to drive diversity within their sports coverage. *The Outer Sanctum* found itself with coveted mainstream media opportunities such as being invited by Melbourne broadsheet daily newspaper *The Age* to cover the first season of the AFLW (national Australian rules women's competition) and from 2017 to 2021, the podcast was produced and distributed by Australia's national broadcaster, the *ABC*.

As the podcast developed to cover both the men's and women's elite Australian rules football seasons, the show continued to encourage difficult conversations about language, inclusiveness, and the wider social impacts of the game. Most notably, the podcast first coined the term “AFLM” to bring attention to the problematic nature of creating a women's product by adding a “W,” while leaving the men's iteration untouched and therefore portrayed as the “real” or “normal” version of the competition.

In an article penned for *The Guardian, The Outer Sanctum* co-founder Seear wrote, “These tendencies in language—to privilege men and erase or omit women—are pervasive, widespread, and continue to linger today (Seear, 2020).” The use of “AFLM” when referring to the men's competition is now widely used within the women in sport and women's sports loving communities in Australia and is an identifier of membership to, and support of, this community.

The other expression the collective adopted was a “nothing about us, without us” approach. Synonymous with movements to amplify consideration and input from persons living with a disability (see Charlton, 1998), the expression acts as a reminder to acknowledge the voices in the room, and more importantly, the voices not in the room when discussing issues or making decisions that impact the lives of others outside of your own lived experience. In this regard, *The Outer Sanctum* took proactive steps to engage with guests, experts and community representatives in episodes discussing issues outside of their lived experience as a group of white, cis-het women.

In 2020, *The Outer Sanctum* made the conscious and strategic decision to evolve from just using the “nothing about us without us” mantra in a special guest format, to move from a group of six to ten, adding Shelley Ware, Tess Armstrong, Julia Chiera and Rana Hussain to the line up to include voices to represent Indigenous Australians, the LGBTIQA+ community and include multicultural and religious diversity. Journalist Kirby Fenwick expresses this audience consideration as, “[t]he community that exists around [*T]he Outer Sanctum* is as original and distinctive as the podcast itself. For the team, the audience factors into a lot of their thinking. Who they are and what they want or need” (Fenwick, 2021 in *Siren Sport*).

For Hussain—a proud Muslim woman of Indian descent and someone experienced in the patience required to see such change occur for more diversity in appointments, this move was meaningful. In an article for *Siren Sport*, Hussain, a diversity and inclusion in sport expert and media commentator, reflected on the invitation to join the team:

“[They were] very clear about the fact that they were a group of white women and wanted to make sure that they were representing the community as best they could. And so instead of going, ‘oh, well, there's already six of us, we'll just have to wait.” They went “no, let's just open up our doors and invite more people in to be part of our group'.” (Hussain in *Siren Sport*, 2021)

This active intersectional inclusion and “opening of doors” that Hussain speaks of is exemplary intersectional inclusion that is rarely seen in sport or sports media. The myth that is perpetuated is that one must patiently wait for an opening, for someone to leave a position, and be in the right place and the right time. But the environment that surrounds alternative and independent media allows for a flexible and innovative approach to inclusive practice. Rather than keep things as they were and maintain that *The Outer Sanctum* is and therefore must remain only six people, they took steps to change their composition to be more reflective of the community they connect with and represent.

*The Outer Sanctum* as a media product and platform still faces challenges and continues to grapple with the best ways to approach sensitive and complicated issues while also maintaining their own identity in the sport media space. This was a reason cited for the decision to amicably part ways with the *ABC* in 2021. Co-founder Lucy Race detailed her thoughts on returning to the independent media space for *Siren Sport*.

“The thing that we are so committed to is always staying true to trying to be a voice for people in the outer and trying to elevate the voices of people in the outer. It's actually a big reason why we've gone back to being independent.” (Race in Siren Sport, 2021)

Race indicates an interesting challenge for the development of intersectionality within mainstream sport media. While it is positive that platforms such as the national broadcaster (*ABC*) and *The Age* engaged with *The Outer Sanctum* to amplify more diverse content, ultimately bringing in an independent platform into mainstream media systems didn't fully allow for the original vision and spirit of the show to continue. For Race, she articulates this return to independence as “[wanting] to be able to speak our truth,” continuing to say that “independence gives us a way to cover football and to cover especially the AFLW, which we love with our whole hearts, in a way that we really feel honours it.” (Race in *Siren Sport*, 2021). Hussain goes on to say the move back to an independent platform will see “deeper conversations, that extended nuance and really grappling with issues.” (Hussain in *Siren Sport*, 2021). These comments indicate that in a mainstream media partnership, these styles of sports coverage were not possible. It can be assumed that in this setting, the show had to adopt the style, standards and practices of a mainstream media organization which has the potential to lead to the issues highlighted previously in the pressure to drive audience and revenue for a broadcaster and cater to a mainstream media strategic vision (Nelson, 2019).

This creates a specific conundrum in the drive for more intersectional content, and content creators, in mainstream sports media as while sports newsrooms maintain standard practices and routine newswork (Sherwood et al., 2017), bringing in and/or partnering with independent media who can assist with diversification can require complicity with structures that have not historically embraced and championed intersectionality.

## Discussion

As we see in the case study of *The Outer Sanctum* podcast, there is a trend that exists alongside the growth of digital media allowing more independent and alternative media offerings to enter the sports media landscape. Many alternative sports media platforms that have been created to challenge or redress historical and current exclusions of coverage and voice in sports media also hold themselves accountable in a way we do not see in mainstream media to be more reflective of the communities they are championing. This can create a catch-22 for these independent content creators as, with most independent media platforms creating content for free or little cost that may only involve small operating funds sourced through crowd-funding platforms or small sponsorships, the work to champion intersectionality and diversity is also continually done by mostly unpaid labor from representatives from marginalized communities. For *The Outer Sanctum* team, this is a key consideration as Race puts it, “we are all trying to fit in something alongside other jobs. We do need to be able to financially support ourselves and to support what we're doing. It is a passion project” (Race in *Siren Sport*, 2021) but also notes that “it is really important to us that we're able to pay people who do work for us, because if we all keep working for no money, then it's just, it's not viable. We need to find ways to make it sustainable” (Race in *Siren Sport*, 2021). Thus, as an independent media platform, *The Outer Sanctum* must navigate both the additional labor of ensuring they are representing their audience through inclusive practice and elevating diverse voices, while finding ways to monetise their product. This commitment to sustainability of their venture and remunerating those involved ensures the labor of diversity does not impact those doing the work to educate, and drive diversity, but commitment to this cause is also labor intensive.

In the case of *The Outer Sanctum*, over time the group has been able to leverage partnerships with mainstream media and the group's members have been offered opportunities to write columns and articles for various publications, provided with guest speaking opportunities and other media appearances. As such, the unpaid labor of the podcast production can lead to additional opportunities, but as highlighted in the case study, these opportunities are fragmented and short-term. Reflecting on this, we must ask how then, can media and organizations use the important intersectional work being done by independent sports media to drive change and implement more intersectional practice within mainstream media in a meaningful, long-term way that sustains the efforts of those creating the content? What our case study suggests is that this cannot be achieved as when this content is brought into the current mainstream media platforms, they are instead shaped and governed by existing rigid media practices that alternative media find challenging to replicate and can feel morally obligated to refuse to perpetuate.

## Conclusion

While challenges exist for independent sports media to continue their work in driving diversity, *The Outer Sanctum* podcast as a media product has produced significant cultural impact on a specific sporting community. This is a community that loves Australian rules football, has felt ignored and/or actively excluded in mainstream media coverage of Australian rules football, and also includes those new to Australian rules football by wanting to support the women's competition and women in sport media. What *The Outer Sanctum* podcast has created goes beyond the show, they have created their own community and connected intersecting communities within football fan spaces, both AFLM and AFLW as well as the amateur and community sporting space. This kind of impact needs further investigation and consideration to determine the extent of its capacity to drive change as currently such media products and content are not given attention and indeed, the respect they deserve when they are continually challenged to not be “real” journalists or part of the mainstream media (Sherwood, 2019).

Yet the case study also highlights significant existing limitations that should be addressed and sought to overcome. These include limited resources and capital, an over-reliance on volunteerism, the potential for burnout (Sherwood, 2019), and for those adopted and embraced by mainstream media—the possible manipulation or corruption of the product to make it more representative of “mainstream” society or to fit within the existing rigid conventions of mainstream sports media and systemic organizational cultures. From this scoping investigation, it appears that until the focus shifts from placing diverse voices into existing structures that perpetuate a routinized (Sherwood et al., 2017) and inflexible approach to content creation, style and dissemination, genuine intersectional media representation and practice cannot be achieved and intersectional representation in sports media will continue to remain in the outer *via* independent and alternative media platforms.

## Data Availability Statement

The original contributions presented in the study are included in the article/supplementary material, further inquiries can be directed to the corresponding author/s.

## Author Contributions

KS prepared the case study and discussion. SD prepared the introduction and literature review. ES provided narrative construction to connect key ideas and arguments throughout the article, reviewed related literature, and recent research. All authors contributed for providing concluding arguments to develop the conclusion. All authors contributed to the article and approved the submitted version.

## Conflict of Interest

The authors declare that the research was conducted in the absence of any commercial or financial relationships that could be construed as a potential conflict of interest.

## Publisher's Note

All claims expressed in this article are solely those of the authors and do not necessarily represent those of their affiliated organizations, or those of the publisher, the editors and the reviewers. Any product that may be evaluated in this article, or claim that may be made by its manufacturer, is not guaranteed or endorsed by the publisher.
